# Facilitating implementation of primary care mental health over time and across organizational contexts: a qualitative study of role and process

**DOI:** 10.1186/s12913-023-09598-y

**Published:** 2023-06-01

**Authors:** Mona J. Ritchie, Louise E. Parker, JoAnn E. Kirchner

**Affiliations:** 1 VA Behavioral Health Quality Enhancement Research Initiative (QUERI), Central Arkansas Veterans Healthcare System, 2200 Fort Roots Dr, North Little Rock, AR 72114 USA; 2 Department of Psychiatry and Behavioral Sciences, College of Medicine, University of Arkansas for Medical Sciences, 4301 W. Markham St, Little Rock, AR 72205 USA; 3 Department of Management, University of Massachusetts, 100 Morrissey Blvd, Boston, MA 02125 USA

**Keywords:** Implementation facilitation, Implementation research, Primary care, Mental health, Integrated primary care, Evidence-based program

## Abstract

**Background:**

Healthcare organizations have increasingly utilized facilitation to improve implementation of evidence-based practices and programs (e.g., primary care mental health integration). Facilitation is both a role, related to the purpose of facilitation, and a process, i.e., how a facilitator operationalizes the role. Scholars continue to call for a better understanding of this implementation strategy. Although facilitation is described as dynamic, activities are often framed within the context of a staged process. We explored two understudied characteristics of implementation facilitation: 1) how facilitation activities change over time and in response to context, and 2) how facilitators operationalize their role when the purpose of facilitation is both task-focused (i.e., to support implementation) and holistic (i.e., to build capacity for future implementation efforts).

**Methods:**

We conducted individual monthly debriefings over thirty months with facilitators who were supporting PCMHI implementation in two VA networks. We developed a list of facilitation activities based on a literature review and debriefing notes and conducted a content analysis of debriefing notes by coding what activities occurred and their intensity by quarter. We also coded whether facilitators were “doing” these activities for sites or “enabling” sites to perform them.

**Results:**

Implementation facilitation activities did not occur according to a defined series of ordered steps but in response to specific organizational contexts through a non-linear and incremental process. Amount and types of activities varied between the networks. Concordant with facilitators’ planned role, the focus of some facilitation activities was primarily on doing them for the sites and others on enabling sites to do for themselves; a number of activities did not fit into one category and varied across networks.

**Conclusions:**

Findings indicate that facilitation is a dynamic and fluid process, with facilitation activities, as well as their timing and intensity, occurring in response to specific organizational contexts. Understanding this process can help those planning and applying implementation facilitation to make conscious choices about the facilitation role and the activities that facilitators can use to operationalize this role. Additionally, this work provides the foundation from which future studies can identify potential mechanisms of action through which facilitation activities enhance implementation uptake.

**Supplementary Information:**

The online version contains supplementary material available at 10.1186/s12913-023-09598-y.

## Introduction

Scholars have long agreed that although the implementation of evidence-based practices and programs will improve the quality of healthcare [1–3], the process of implementing and sustaining them is challenging [4–6]. This may be especially true for integrating mental health services into primary health care settings, termed primary care mental health integration (PCMHI) in this paper. PCMHI models are complex and implementing them requires structural and process changes to care delivery [7–9]; changes in providers’ roles, skills, values, and attitudes [8, 10–12]; and changes in organizational culture [13, 14] and work processes [8, 11]. Additionally, the characteristics of primary care settings, including their capacity for change, vary extensively. Although some settings may be able to implement such complex programs on their own, many lack the infrastructure, resources, skills, or a combination of these characteristics [4]. One implementation strategy, facilitation, has been successfully utilized in research and large-scale clinical initiatives, particularly in primary care settings, to address such challenges and support implementation of evidence-based innovations, including PCMHI [9, 15–22].

Although facilitation as a helping process has been utilized for a variety of purposes, its application for supporting implementation has been described as a “multi-faceted interactive process of problem solving, enabling and supporting individuals, groups, and organizations in their efforts to adopt and incorporate innovations into routine practices” [23]. It is both a role, related to the overall purpose of facilitation, and a process (i.e., how a facilitator operationalizes the role) [24, 25]. Not surprisingly, given the challenges to implementation and sustainment of evidence-based innovations such as PCMHI, the process of facilitation is complex. Facilitators supporting implementation often conduct a wide variety of activities [25–27]. For example, they typically assess the clinical setting and organizational context for implementation and engage stakeholders who will be responsible for implementing the innovation, supporting its implementation, or receiving it. They may also work with site stakeholders and teams to develop an implementation plan, assess implementation progress, identify and address challenges, and provide ongoing support for implementation. Although studies have been documenting the range of activities facilitators perform, scholars continue to call for a better understanding of what facilitators do [28, 29].

Scholars agree that facilitation is a dynamic process, but little is known about its dynamic nature. Detailed descriptions of what facilitators do are often framed within the context of a staged process. Dogherty and colleagues created a taxonomy of facilitation activities organized around “stages” of facilitation (i.e., planning for change, leading and managing change, monitoring progress and ongoing implementation, and evaluating change); others have continued to build upon this framework [26, 30–32]. Although the integrated Promoting Action on Implementation Research in Health Services (i-PARIHS) framework assumes that implementation is non-linear and complex and posits that facilitation tailors the implementation process in response to the innovation, its recipients, and the implementation context, the framework describes facilitation activities in sequential “phases” (i.e., clarifying and engaging, assessing and measuring, action and implementation, and reviewing and sharing) [25]. Describing what facilitators do as a staged process may make it easier to plan for facilitating implementation. In reality, we have little understanding of how the implementation facilitation process changes over time and in response to context.

Another understudied characteristic of the facilitation process is related to the relationship between the role of facilitators and how that role is operationalized through activities. The facilitator’s role depends on the purpose of facilitation. Scholars propose a “facilitation continuum.” On one end of the continuum, the purpose of facilitation, and thus the role of the facilitator, is task-focused, e.g., to implement an evidence-based practice or program [33, 34]. On the other end of the continuum, the purpose of facilitation, and thus the role of the facilitator, is holistic, e.g., to develop and empower individuals and teams and create a supportive context for change [29, 33, 35, 36]. The intended role of a facilitator can lie anywhere along this continuum. Additionally, scholars have suggested that the activities facilitators conduct to operationalize their roles also lie on a continuum. On one end, facilitators are doing particular activities for individuals and teams; at the other end of the continuum, facilitators are enabling individuals and teams to do activities for themselves [36]. We know little about the balance between different facilitation activities and the actual facilitation role [37].

This study was part of a large project that successfully tested a facilitation strategy within the context of a Department of Veterans Affairs (VA) national initiative to implement evidence-based PCMHI care models [38]. The facilitation strategy was informed by the original Promoting Action on Implementation Research in Health Services (PARIHS) framework [33, 39]. The project utilized a two-person facilitation team consisting of an expert external facilitator (EF) and an internal regional facilitator (IRF). The EF provided expertise about facilitation and supervised and supported development of the IRF’s skills and knowledge related to facilitation and managing change. The IRF provided specific institutional knowledge and access. The purpose of this facilitation strategy was both task-focused (i.e., to support PCMHI implementation) and holistic (i.e., to build capacity for future implementation efforts). Previous studies related to this project demonstrated that this strategy increased the reach and adoption of PCMHI [40] and improved PCMHI program uptake, quality and adherence to evidence [41]. As in those papers, we call the facilitation strategy *implementation facilitation* to emphasize that the purpose of facilitation was to support implementation of PCMHI. The present study had two goals. First, we explored how the implementation facilitation process changed over time and in response to context so as to better understand how facilitators foster system change. Related to this goal, we explored how facilitators operationalized their role and how their activities varied from doing for others to enabling others to do for themselves.

## Methods

### Study design, setting, and participants

We explored the facilitation process using a qualitative descriptive study design [42, 43]. Such methods foster the conduct of in-depth exploration and discovery of new knowledge. By their nature, they are labor intensive and expensive, and thus must sacrifice the large sample sizes needed for study breadth in favor of the deep study of a small sample [44].

When we conducted the larger project, VA was comprised of 21 geographic regions called networks. We selected and recruited two networks (A and C) based on 1) strength of the mental health leadership structure, 2) ability to identify an internal regional facilitator who could devote 50% effort to facilitate the clinical initiative, and 3) willingness to participate. Participating network mental health leaders identified four primary care clinics, one located in a VA medical center and three in community-based outpatient clinics that 1) would have difficulty implementing PCMHI without assistance and thus would benefit from facilitation, 2) served, or had potential to serve, 5000 or more primary care patients, and 3) planned to implement a PCMHI program. All eight clinics in Networks A and C received facilitation to support PCMHI implementation.

Study participants included the expert EF and two IRFs (i.e., one for each of the four Network A clinics and one for each of the four Network C clinics). The EF (JEK) was a psychiatrist with expertise in PCMHI care models, implementation science, facilitation, and mentoring. One of the IRFs was a doctoral level psychologist who had been a mental health therapist and educator. The other IRF was a master level social worker who had clinical training and extensive experience in program quality improvement. Both IRFs were network level employees who initially had no implementation science or implementation facilitation expertise.

### Data collection

Two highly experienced qualitative researchers (LEP and MJR) conducted individual monthly one-hour long debriefings by telephone with the three facilitators over the 30-month intervention period from June 2009 to November 2012. Both interviewers had long-term professional relationships with the expert (JEK). The purpose of the debriefings was to track the on-going facilitation process at each site and identify contextual factors and events that might foster or hinder PCMHI implementation. The EF debriefings also provided information on the facilitation process and relevant events but focused on the process she used to coach, train, and mentor the internal facilitators. The latter is the subject of other analyses [45, 46]. All debriefings used a semi-structured format. In total, we conducted 85 debriefing interviews.

The first author (LEP), an organizational scientist, acted as the primary interviewer and the second author (MJR), an implementation scientist, acted as a secondary interviewer, asking back up questions and ensuring that all major topics were explored fully. Both interviewers took detailed notes and documented facilitators’ responses as close to verbatim as possible. MJR then wrote up summary notes which LEP reviewed. The two interviewers discussed and resolved any differences in their observations; where they were unable to reach consensus, they followed up with the facilitator during the next debriefing to obtain clarification.

The VA Central Institutional Review Board CIRB; (#09–05) approved the conduct of the larger project, which was conducted between February 2009 and August 2013. Their approval included the documentation of facilitators’ quality improvement activities through debriefing interviews without a formal informed consent process.

### Data analysis

Interviewers conducted a targeted search of facilitation literature to identify literature that described activities facilitators performed. To create a list of activities with definitions, they conducted a content analysis of activity definitions in selected articles and combined those that used different labels but described the same actions. Next, interviewers reviewed the debriefing notes to determine if there were any additional activities that study facilitators performed that were not previously identified and added those to the activity code list. See Additional file 1 for the list of activities, their descriptions, and the source (literature and/or debriefing notes).

Interviewers then conducted a content analysis [47] of the debriefing notes. Using the activity list, they coded the debriefing notes for each clinic by quarter. Coding consisted of 1) identifying which facilitation activities occurred in each quarter; 2) rating the level of intensity (high, moderate, or minor) of each activity in each quarter; and 3) documenting whether facilitators were “doing” these activities for sites, “enabling” site members to perform these activities themselves, or doing a combination of the two. They also documented clarifying comments about the activity and contextual factors to aid with interpretation (see Additional file 2 for the coding template).

To ensure consistency of their coding, both interviewers independently coded the first quarter for all sites in both networks. They then discussed and resolved differences, as well as refined definitions in the activity list. They continued this process through the fourth quarter, by which time their coding had become uniform. They then alternated coding by network for the remaining six quarters. Thus, both interviewers coded all sites in both networks for quarters one through four, one interviewer coded all sites in one network for quarters 5, 7, and 9 and the other network for quarters 6, 8, and 10.

Once completed, they entered data into an Access database, exported this data to Excel spreadsheets, and created tables of activities and their intensity by approximately six-month periods. To explore variation in doing versus enabling, for each network, they divided the number of times they had coded an activity as doing or enabling by the total number of times they coded the activity. Looking for patterns, they created summaries, aggregating across clinics within the two networks so they could compare them.

## Results

### Facilitation activities

We created a comprehensive list of implementation facilitation activities based on our literature review and our debriefing notes (see Additional file 1). We found that our facilitators engaged in all of these with some caveats, as well as some that previous researchers had not identified and that may be specific to our context.

### Facilitation over time and across sites and networks

  Certain implementation facilitation activities tended to occur predominantly during particular implementation periods (see Table 1). Those that occurred primarily at the beginning, were ones that logically either only could (i.e., baseline data collection) or should (e.g., planning) occur during the beginning of the implementation process. Ever evolving context, rather than implementation phase, however, dictated the presence and intensity of most activities at particular times and in particular places. This included many activities that we logically might have expected to occur at the beginning (e.g., task orientation, goal and priority setting), as well as others that have no expected pattern with respect to timing (e.g., providing updates and feedback; see Table 2).

**Table 1:** Activities with highest intensity during particular implementation periods

Activity occurring primarily during the	Notes
**Beginning**	
Baseline data collection	Exclusively at the beginning
Technical Assistance IT Marketing Strategy policy development	Concentrated at the beginning but continued as needed
Action/implementation planning	Recurred if original plan did not fit or with staff changes
Overcoming resistance to change	Concentrated at the beginning but longer at some sites, particularly where local medical center management was not supportive of PCMHI
**Middle**	
Managing team processes	Occurred throughout but highest during the middle when a PCMHI learning collaborative was most active
Fostering networking with peers	Primarily through a PCMHI learning collaborative that did not start until the middle and dissipated over time
**Middle and End**	
Clinical skills education	
Pulling back and letting sites take the lead	By definition was something that happened at the end, but the external facilitator began this process earlier than the internal regional facilitator, by design

*PCMHI* Primary care mental health integration

**Table 2:** Activities where intensity varied based on site needs rather than time periods

**Activities expected to occur primarily at the beginning but did not**
Adapting program to local context
Developing shared vision
Engaging stakeholders
Fostering organizational structural change and organizational cultural change
Goal/priority setting
Problem identification
Task orientation
**Activities with no expected pattern with respect to time**
Attending, presenting at, and organizing regional meetings
Attending and presenting at national meetings
Data collection to conduct on-going monitoring of program implementation
Facilitator continuing education
Fostering contact with experts
Helping to hire clinical program staff
Interceding and liaising with leaders and departments
Marketing education
Problem solving
Providing updates & feedback
Technical assistance/non-IT

There were also systematic differences across sites between the two networks. First, there were a number of activities (providing updates and feedback, providing support, and interceding and liaising with leaders and other departments) that were more common in Network A than Network C. Second, there were a number of activities that occurred at a very low level (marketing education, fostering structural and cultural change) or were completely absent (organizational change skills education) in Network C. As might be expected from these patterns, overall, there was more activity in Network A than Network C.

### Contextual factors and possible effects on activity use and intensity

There were a number of contextual factors that influenced the use, intensity, and timing of particular activities. Although all sites had been selected because they had challenging contexts; not surprisingly, sites experienced different challenges at different times and some experienced more challenges than others. Below we provide examples of these factors and how they may have influenced what facilitators did to support PCMHI implementation.

#### Site context

##### Turnover

There was turnover at every level in key positions such as clinical leaders and PCMHI providers. When this occurred, facilitators had to return to activities that we would expect to occur early in the process such as task orientation and stakeholder engagement so new staff could be brought up to speed.

##### Lack of high level-leadership support

At one VA medical center, lack of high-level leadership support hindered PCMHI implementation in its primary care clinic and in the associated community-based outpatient clinic by limiting the resources that were needed for structural change. To address this challenge, the facilitator had to spend more time over a longer period in stakeholder engagement and overcoming resistance to change activities.

##### Challenges to transitioning from a traditional mental health service model to PCMHI

Some PCMHI providers struggled to make the transition from a mental health service model with lengthy wait-times, lengthy appointments, and long-term follow-up to a PCMHI model with same-day access, briefer appointment times, and briefer interventions. When this problem was identified, the IRF had to spend more time over a longer period shadowing providers to assess their practice, and providing clinical skills education, mentoring, and support to them, as well as helping site supervisory staff identify ways that they could support struggling providers.

##### Competing demands

At many of our sites, when there was turnover and resulting staff shortages in the mental health service clinics, PCMHI providers were expected to provide traditional mental health services. Whenever this occurred, facilitators helped them identify solutions. For example, one small clinic lost one of their traditional mental health providers and there were no plans to replace him. The facilitator helped the PCMHI provider modify his role so that he provided both brief PCMHI type treatment as well as a modified version of specialty mental health treatment to help the short-staffed mental health clinic minimize their wait-times.

#### Network context

There were also differences in organizational context at the network level. At the start of the study, Network A had already adopted a model of PCMHI and infrastructure to support it. However, the model was not compliant with VA national requirements. Network C was not as familiar with the concept of PCMHI, and they lacked the infrastructure support for implementation. Thus, Network A sites were generally more receptive to the general concept, although not initially the particulars of PCMHI than were those in Network C, and the Network A facilitator engaged more in the day-to-day work of collaborating with sites on implementing the program than the Network C facilitator. Interestingly, the Network C facilitator did engage more in attending, organizing, and presenting at regional meetings than the Network A facilitator.

### Doing versus enabling

Concordant with the planned facilitation role, we found that facilitators engaged in both “doing” for sites as well as “enabling” sites to engage in a variety of activities for themselves (see Table 3). Facilitators primarily did some activities for sites, i.e., activities were toward the “doing for” end of the continuum. As might be expected, these activities fell into one or more of the following categories: 1) site members would have found them burdensome, 2) site members lacked the requisite skills to perform them (e.g., data collection), and 3) involved recruiting support for change or providing education. Facilitators primarily “enabled” sites members to do other activities themselves, i.e., these activities were more toward the “enabling” end of the continuum. Also, as might be expected, activities that primarily involved enabling generally require site members’ active participation to be successful (e.g., developing a shared vision and consensus and adapting program to local context). Many activities did not fall primarily toward one end of the doing/enabling continuum. For example, in Network A, facilitators did Marketing for sites 50% of the time and enabled site members to market for themselves 50% of the time. In terms of the continuum, in this network, the Marketing activity was in the middle of the continuum. Further, the extent to which some activities were “doing” versus “enabling” varied by network. See Table 3.

**Table 3:** Network variation in extent to which activities were doing versus enabling

**Primarily “doing for others” across networks**		
Conduct ongoing monitoring of program implementation		
Clinical skills education		
Data collection to assess baseline practices		
Engaging stakeholders		
Managing team processes		
Project management/administrative tasks		
Providing support		
Providing updates and feedback to project participants		
Task orientation		
Technical assistance		
**Primarily “enabling others to” across networks**		
Action/implementation planning		
Adapting program to local context		
Developing shared vision/consensus building		
Fostering organizational change		
Fostering peer networking and contact with experts		
Problem identification		
Problem solving		
**“Doing for” versus “enabling others to” was network dependent**		
Activity	Network A	Network C
Change skills education	100% Doing	No involvement
Goal/priority setting	57% Enabling	67% Doing
Helping to hire clinical program staff	50% Doing/Enabling	No Involvement
Interceding and liaising with leadership and other departments	65% Enabling	50% Doing/ Enabling
Marketing	50% Doing/Enabling	100% Doing
Marketing Education	50% Doing/Enabling	No Involvement
Overcoming resistance to change	73% Doing	60% Enabling
Strategy/policy development	50% Doing/Enabling	67% Enabling
Technical assistance/ IT	50% Doing/Enabling	62% Enabling

## Discussion

Previous studies related to the larger project demonstrated that implementation facilitation increased the reach and adoption of PCMHI in VA primary care clinics [40] and improved PCMHI program uptake, quality and adherence to evidence [41]. The current study explored characteristics of the facilitation process. It focused on how the use and intensity of particular facilitation activities changed over time and in response to context. Our findings fill a gap in the literature regarding characteristics of the facilitation process. Because facilitation is being increasingly utilized to support implementation of complex evidence-based practices and programs, improving our understanding of this process can maximize the potential for supporting innovation implementation in complex settings (i.e., primary care) and building capacity for change.

Perhaps our most significant finding concerned the timing of activities. Facilitation activities did not occur according to a defined series of ordered steps or phases as some scholars have described [25, 30]. In fact, for most activities, there was no pattern to their occurrence. Rather, consistent with many organizational change scholars [48] and a growing number of implementation scientists [28, 49], activities occurred in response to specific organizational contexts through a non-linear and incremental process. To our knowledge, only one other study has explored temporal patterns of facilitation activities. This study found patterns in timing of types of activities with some occurring most frequently early in the process [29]. Our study, which explored discrete activities rather than types, suggested that intensity of some activities within these types was higher early in the process but for others, there was no expected pattern. For example, Baloh and colleagues found that engaging stakeholders, a part of their ‘leadership’ type, occurred most frequently early in the implementation process [29]; we found that the intensity of this activity varied based on site need rather than time period. The difference in our findings may be related to the complexity of PCMHI and the primary care environment, the high staff turnover rate, or the approaches to exploring activities (e.g., types versus discrete activities). Regardless, our findings support the supposition that facilitation is a dynamic and fluid process [36]. Facilitators and other implementation support agents need to be aware of the wide variety of activities for which there is no expected pattern of intensity. It may be that patterns of activities in facilitation phase models are based on when activities start rather than when they are provided most intensely. Future research should explore this.

Although we also found that our facilitators participated in virtually all activities that previous researchers had identified, as well as several previously unidentified ones, we identified substantial variation between the two networks participating in the study. These differences were both in types of activities that predominated and the overall amount of activity that occurred. One possible explanation for these findings concerns variation between the networks’ responsiveness to PCMHI and the available infrastructure support for it. Because Network A sites were generally receptive to PCMHI and had existing infrastructure support at the network level, the facilitator was able to collaborate with local sites directly to implement the program. In Network C, where sites were less familiar with PCMHI and there was no infrastructure support, the facilitator engaged more in attending, organizing, and presenting at regional meetings than the Network A facilitator, possibly in an effort to change regional attitudes towards the program. It might have also been easier and more rewarding to engage in regional level activities than site level ones as the facilitator was experiencing so much local push back.

Implementation scientists have suggested that certain characteristics and skills shape what facilitators do in the field and their ability to be successful [29]. In fact, there were several differences between the two IRFs that could help explain some of the differences in their activity. For example, the Network A IRF was a skilled educator and most comfortable working one-on-one with staff to effect change; the Network C facilitator had substantial experience working in re-engineering and was most comfortable operating at the system level. Ultimately, we cannot be sure of the extent to which differences in the two networks were due to contextual factors or the facilitators themselves. Although their actions were appropriate to their contexts, they also matched the facilitators’ own strengths. Regardless, our findings confirm that the process of facilitation includes selecting activities that will address the needs of the organizational context [50, 51]. However, activities facilitators select may also be influenced by their skills and other characteristics.

Finally, the purpose of implementation facilitation in this study was both task-focused and holistic. Concordant with this purpose and their intended roles, facilitators conducted some activities primarily focused on doing things for sites and others focused primarily on enabling sites to do things for themselves. We also found that some activities could be either something that the facilitator “did” for or “enabled” the clinic to do for themselves, depending on needs, skills, and other organizational circumstances. For example, as Network A sites were generally more amenable to the general concept of PCMHI than were Network C sites, in Network A facilitators both did marketing for and enabled sites to do their own marketing; whereas in Network C, facilitators did all the marketing for sites. Similarly, as Network C lacked infrastructure support for implementation, facilitators focused more than Network A facilitators on enabling sites to develop policies and the infrastructure needed to support PCMHI adoption. When the focus of an activity varied between doing and enabling, it is also possible that facilitators were initially doing the activity for sites in order to model how it was done and then later enabling the site to do this activity on their own [46]. Alternatively, it is possible that, as others have suggested, it was easier at times for facilitators to slip into doing activities rather than enabling the site to do them [52, 53].

Understanding the relationship between the role of facilitators and how that role is operationalized through activities has implications for those planning or applying an implementation facilitation strategy. When planning a facilitation strategy, an initial decision about the purpose of facilitation and where it lies on the task-focused/holistic continuum can guide planners in deciding which activities they want facilitators to conduct to operationalize this purpose, as well as which skills facilitators will need and perhaps how facilitators might be supported to maximize the potential for achieving this purpose. For example, if the purpose of facilitation is partially or entirely holistic, then facilitation activities selected to operationalize the related role must include a large measure of enabling activities. Even for activities that were primarily done for sites in our study, i.e., providing support, facilitators may need to focus more on enabling site members to provide support for themselves. Thus, facilitators will need the knowledge and skills to be able to flexibly move along the doing for/enabling to continuum with a goal of sites ultimately being able to take responsibility for facilitation activities. If facilitators don’t have the needed skills and experience, it may be important to plan to provide mentoring, as we did, so that a more experienced facilitator can model how to conduct activities along the continuum.

When applying a facilitation strategy, understanding characteristics of the facilitation process may help facilitators to stay focused on the intended purpose of their efforts and how they might best operationalize that purpose. For example, if the purpose is partially or entirely holistic and the intent is for site members to conduct facilitation activities for themselves, facilitators, or those responsible for them, will need to monitor how they are conducting activities and attempt to address any inclination they might have to “do for” rather than “enable to” or favor activities that are more easily done for site members [53]. In addition, understanding the relationship between the purpose of facilitation and how it is operationalized through activities conducted on a doing for/enabling to continuum can inform evaluations of facilitation strategies. Perhaps most important, better understanding of this process can provide the foundation upon which an understanding of why, or why not, facilitation activities are successful and the mechanisms upon which the activities enact change [54].

Additionally, Proctor and colleagues provided guidance on how implementation strategies should be specified and reported to ensure comparability across studies and accelerate our understanding of how they work [55]. We explored two of their dimensions for describing the operationalization of strategies: action (the activities facilitators conducted) and temporality (i.e., the order or sequence of strategy use across the phases of implementation). In this study, activities did not occur according to a defined series of ordered steps or phases but were responsive to context. Given the need for facilitators to adapt what they do to local context [50], reporting on the sequence of activities could be challenging and perhaps not helpful. It may be that there are other dimensions of implementation facilitation strategies that should be specified and reported. In this study, we explored the relationship between the facilitation role and facilitation activities. The planned role, on the continuum from doing to enabling, may be one of those dimensions. There may be other dimensions of implementation facilitation that need to be specified, applied, and evaluated. Future work should identify these.

This study has several limitations. Both the particular clinical context, primary care, and the evidence-based program being implemented, PCMHI, are highly complex. The implementation of many evidence-based practices, however, does not require such fundamental changes. For example, changing the type of medications that providers use for managing cholesterol levels may not require the entire set of implementation facilitation activities we have identified. Further, given that we found that context plays such an important role in implementation facilitation, our findings might have varied if we had examined clinics within different VA networks, different VA clinics within the same networks, or clinics within different health systems within the US and in other countries.

## Conclusions

Our findings are a first step but are intriguing enough, we believe, to suggest that further longitudinal research across a broad range of clinical and organization contexts would help us deepen our understanding of how facilitation fosters evidence-based practice implementation. And in doing so, we may be able to more specifically target interventions to particular contexts or to effectively design and tailor implementation strategies. In addition, this work provides the foundation from which future studies can identify the potential mechanisms of action through which facilitation activities enhance implementation uptake of an evidence-based practice or program.

## Supplementary Information

**Additional file 1.** Facilitation Activities in Literature and Debriefing Notes.

**Additional file 2.** Debriefing Interviews Coding Template.

## Data Availability

The data generated during this study are not publicly available because facilitators were assured prior to consent that information they provided would not be publicly available and institutional restrictions prohibit the sharing of protected data. Data may be available from the corresponding author upon reasonable request and with the permission of the Department of Veterans Affairs.

## Abbreviations

EF: External facilitator
IRF: Internal regional facilitator
PCMHI: Primary care mental health integration
VA: Department of Veterans Affairs
