# Supplementary material for: Facilitating implementation of primary care mental health over time and across organizational contexts: a qualitative study of role and process
Source: BMC Health Serv Res. 2023 Jun 1;23:565. doi: 10.1186/s12913-023-09598-y (PMC10233920; doi:10.1186/s12913-023-09598-y)
Supplement: Supplementary file 1 — Additional file 1. Facilitation Activities in Literature and Debriefing Notes. [file 12913_2023_9598_MOESM1_ESM.docx]

**Additional file 1**

**Facilitation Activities in Literature and Debriefing Notes**

| **Activities identified in both literature and debriefing notes:** | **Description based on literature and/or debriefing notes** |  |
| --- | --- | --- |
| Action/implementation planning [1-8] | Assisting with development of Action Plans/Implementation Plan including both formal action items, short term plans and long-term formal plans/implementation checklist. |  |
| Adapting program to local context [2, 5, 9-12] | Helping to adapt to and create synergy with local context, including local structure, staffing, culture, and other initiatives. |  |
| Assisting with hiring staff internal regional facilitators^†^ [9] | Helping select and hire internal facilitator, includes helping with writing job descriptions. |  |
| Clinical skills education [2, 5-25] | Providing education/information about clinical content skills/expertise, includes provider education, clinical training/supervision/ coaching & mentoring, academic detailing (can be ongoing). Also includes counseling, co-counseling, critical reflection, new ways of work, experiential learning, learning from practice.  *This does not include presentations at regional and or national meetings.* |  |
| Data collection to assess baseline practices [2, 7-9, 17, 20, 21, 23, 26] | Collecting/reviewing numerical diagnostic information/data to understand the local context, baseline performance. |  |
| Data collection to conduct on-going monitoring of program implementation [1, 2, 4-8, 15, 19, 23, 26-28] | Monitoring/tracking/collecting data/information on progress, problems, fidelity to evidence, performance, and PCMHI activities. Includes quantitative and qualitative data, observations, etc. |  |
| Developing shared vision/consensus building [2-5, 7, 8] | Finding synergy between existing goals and PCMHI goals, helping stakeholders to “get on the same page,” helping stakeholders to see what is in it for “me and us,” developing “win-win solutions.” |  |
| Engaging stakeholders, obtaining buy-in [2, 4, 5, 8, 9, 12] | Engaging stakeholders and seeking their participation/buy-in. |  |
| Fostering peer networking [1, 2, 9, 15, 19, 22-24, 26] | Fostering networking, communication across sites, learning collaboratives, cross pollination, using opinion leaders. |  |
| Fostering contact with experts [1, 2, 9, 15, 19, 22-24, 26] | Linking to external expert contacts. |  |
| Goal/priority setting [2-5, 7, 8, 23] | Assisting in setting clear, realistic goals, setting priorities.  “Thinking big but starting small.” |  |
| Interceding and liaising with leaders and other departments [2, 5, 6, 23] | Interceding/liaising with leadership (internal or external) and relevant departments about PCMHI program including Joint Commission issues, facilitation, implementation, or other issues. |  |
| Managing team processes [2, 3, 7, 12, 19, 25, 27] | Includes managing group dynamics; running effective meetings; keeping group focused; establishing team structure, membership, roles, & ground rules; creating atmosphere of mutual respect; enhancing communication with a practice; fostering democratic/participatory process. |  |
| Marketing [2, 5, 6, 9, 11, 15-17, 20, 21, 28] | Includes providing evidence for PCMHI conducted by facilitators. |  |
| Marketing education [3, 5-9, 23, 24] | Providing education about how to conduct marketing to clinic, medical center, and network staff members. Includes teaching PCMHI providers how to market their own skills and services. |  |
| Organizational change skills education [3, 5-9, 23, 24] | Providing education/information about change skills/processes, including skills, incentives, and resources to implement change and information about organizational systems and change processes, evaluation, how to engage stakeholders in change process, empowering participants, ensuring have sufficiently diverse viewpoints except marketing. |  |
| Overcoming resistance to change [2, 3, 6, 8, 14, 16, 25] | Working with leaders and providers (including PCMHI providers) to overcome resistance to PCMHI. This can include convincing them that Primary Care can handle certain mental health disorders safely, that brief treatments & assessments are effective & efficacious for some patients, that PCMHI is primarily a quality of care not cost savings intervention, that PCMHI will increase access to mental health for those who need it and to mental health treatment for all patients, etc.  *This code may overlap frequently with: Engaging stakeholders, Marketing, and the various education codes. It might also overlap with organizational change codes. This code focuses more on individual attitudes than on shared beliefs among members of an organization or sub-groups within an organization*. |  |
| Problem identification [1-3, 5, 7, 8, 12, 17-21, 23, 26] | Assisting with problem identification, awareness, & clarification including understanding current ways of working and thinking, current context and identifying gaps & barriers. |  |
| Problem-solving [2, 18, 24-26] | Assistance with problem-solving, brain-storming solutions. |  |
| [Project management] /Administrative tasks^‡^ [2, 16, 17, 20, 21, 28] | Setting up site visits, scheduling and inviting stakeholders to calls and meetings, organizing meetings, emailing announcements and reminders, setting up monthly PCMHI provider calls, creating and sending out meeting minutes, preparing and disseminating reports and materials, scheduling/arranging speakers for monthly calls, making arrangements for PCMHI provider training (e.g., sending providers to other clinics to shadow more experienced providers), preparing PowerPoint slides. |  |
| Providing support [1, 2, 5, 8, 9, 15-17, 21, 23, 27] | Being generally helpful and available, being available for questions, providing encouragement, doing things in a warm, encouraging, and empathetic way rather than hypercritical, punishing way, demonstrating “people skills,” using carrots rather than sticks, etc. |  |
| Providing updates & feedback to project participants [2, 4, 6-8, 13, 15, 19, 23, 26, 27] | Providing updates on implementation, including providing feedback on data, Plan-Do-Study-Act cycles, PCMHI provider activities, facilitator activities, relevant VA and other professional information (e.g., availability of new guidelines, tools, etc.). |  |
| Strategy/policy development [1-8] | Assisting with strategy and policy development. |  |
| Task orientation [1-3, 5, 7, 8, 12, 17-21, 23, 26] | Describing purpose and process of PCMHI, facilitation, what will occur, outlining roles. |  |
| Technical assistance/non-IT and Technical assistance: IT [2, 5, 10, 16, 17, 20-23, 26] | Providing technical assistance (e.g., providing tools and sample materials [e.g., reminder systems, templates, patient materials, etc.]), developing necessary infrastructure to support adoption other than IT and site members and facilitator(s) working on creating documents/evaluation materials together.  Working with IT to ensure that IT systems accurately capture and support PCMHI activity (e.g., encounters) and anything related to computer issues, including software. |  |
| **Activities identified in debriefing notes only:** | **Description based on debriefing notes** | |
| Attending and presenting at national meetings | Attending or presenting at national meetings such as PCMHI, PACT, and National Mental Health meetings. | |
| Attending, presenting at, and organizing regional meetings | Attending, presenting at, or organizing regional PCMHI and PACT meetings as well as Primary Care and Mental Health Councils/Advisory Boards and Center for Integrated Healthcare meetings (not site visits). | |
| Engaging in regional and national spread: clinical content and facilitation methods | Assisting with regional or national spread of clinical content, i.e., PCMHI, or facilitation methods beyond facilitation sites/networks. | |
| Facilitator continuing education | Attending trainings, meetings, webinars, tele- or videoconferences, etc., reading, and obtaining relevant tutorials from experts for purposes of learning about any of the following: facilitation, relevant clinical content, relevant technical skills (e.g., software, data collection methods).  *Excludes training by External Facilitator.* | |
| Fostering organizational change: cultural | Promoting cultural change required for implementation. Changes in shared beliefs about care, types of providers skills and practice scopes (not specific providers, but classes of providers such as primary care providers, social workers, psychiatrists, psychologists, nurses, etc.), efficacy and safety of treatments and practice models. These may be shared beliefs among all members of an organization or members of a sub-group (e.g., mental health providers, nursing, primary care providers, physicians, etc.). | |
| Fostering organizational change: structural | Promoting structural change required for implementation (e.g., staffing changes, reporting structure changes, office assignment changes, methods for referring patients, how patients move through the system [e.g., physically get to PCMHI providers, get from PCMHI providers to front desk], etc.). | |
| Fostering organizational change: unspecified | Promoting organizational change (not specified) required for implementation. | |
| Helping to hire clinical program staff | Assisting with hiring PCMHI providers, replacements, or additions, includes helping with writing job descriptions. | |
| Pulling back and letting sites take lead | Attending regional PCMHI and PACT meetings as well as Primary Care and Mental Health Councils/Advisory Boards and Center for Integrated Healthcare meetings (not site visits). | |

^†^The networks selected their own internal regional facilitators, although the external facilitator provided job descriptions and advice. The Network C internal regional facilitator retired during the last year of the study and both she and the external facilitator helped select a replacement.

^‡^As defined in the literature and in practice, project management (PM) includes goal setting, developing plans, identifying and allocating resources, determining timelines, monitoring performance, providing feedback, and managing budgets. Our facilitators did not control timelines or manage budgets; we classified the remaining PM tasks under other specific tasks. The facilitators also engage in many administrative activities including organizing meetings and preparing reports that are generally subsumed under PM. We coded those as administrative tasks.

References

1. Bidassie B, Williams LS, Woodward-Hagg H, Matthias MS, Damush TM. Key components of external facilitation in an acute stroke quality improvement collaborative in the Veterans Health Administration. Implement Sci. 2015;10(1):69. <https://doi.org/10.1186/s13012-015-0252-y>

2. Dogherty EJ, Harrison MB, Baker C, Graham ID. Following a natural experiment of guideline adaptation and early implementation: a mixed-methods study of facilitation. Implement Sci. 2012;7:9. <https://doi.org/10.1186/1748-5908-7-9>

3. Hayden P, Frederick L, Smith BJ, Broudy A. Developmental facilitation: Helping teams promote systems change. Collaborative Planning Project for Planning Comprehensive Early Childhood Systems. Denver; 2001: 2-21.

4. Helfrich CD, Li YF, Sharp ND, Sales AE. Organizational readiness to change assessment (ORCA): development of an instrument based on the Promoting Action on Research in Health Services (PARIHS) framework. Implement Sci. 2009;4:38. <https://doi.org/10.1186/1748-5908-4-38>

5. Hogg W, Baskerville N, Nykiforuk C, Mallen D. Improved preventive care in family practices with outreach facilitation: understanding success and failure. J Health Serv Res Policy. 2002;7(4):195-201.

6. Nzinga J, Ntoburi S, Wagai J, Mbindyo P, Mbaabu L, Migiro S, et al. Implementation experience during an eighteen month intervention to improve paediatric and newborn care in Kenyan district hospitals. Implement Sci. 2009;4(1):45. <https://doi.org/10.1186/1748-5908-4-45>

7. Thomas P, McDonnell J, McCulloch J, While A, Bosanquet N, Ferlie E. Increasing capacity for innovation in bureaucratic primary care organizations: A whole system participatory action research project. Ann Fam Med. 2005;3(4):312-317.

8. Wright J, McCormack B. Practice development: individualized care. Nurs Stand. 2001;15(36):37-42. <https://doi.org/10.7748/ns2001.05.15.36.37.c3032>

9. Kirchner J, Edlund CN, Henderson K, Daily L, Parker LE, Fortney JC. Using a multi-level approach to implement a primary care mental health (PCMH) program. Fam Syst Health. 2010;28(2):161-174. <https://doi.org/10.1037/a0020250>

10. Lombarts MJ, Klazinga NS, Redekop KK. Measuring the perceived impact of facilitation on implementing recommendations from external assessment: lessons from the Dutch visitatie programme for medical specialists. J Eval Clin Pract. 2005;11(6):587-597.

11. McWilliam CL, Kothari A, Ward-Griffin C, Forbes D, Leipert B, South West Community Care Access Centre Home Care Collaboration (SW-CCAC). Evolving the theory and praxis of knowledge translation through social interaction: a social phenomenological study. Implement Sci. 2009;4(1):26. <https://doi.org/10.1186/1748-5908-4-26>

12. Wallin L, Rudberg A, Gunzerath L. Staff experiences in implementing guidelines for Kangaroo Mother-Care-a qualitative study. Int J Nurs Stud. 2005;42(1):61-73.

13. Burrows DE. Facilitation: A concept analysis. J Adv Nurs. 1997;25(2):396-404.

14. Cross KD. An analysis of the concept facilitation. Nurse Educ Today. 1996;16(5):350-355.

15. Doran DM, Sidani S. Outcomes-focused knowledge translation: a framework for knowledge translation and patient outcomes improvement. Worldviews Evid Based Nurs. 2007;4(1):3-13.

16. Harvey G, Loftus-Hills A, Rycroft-Malone J, Titchen A, Kitson A, McCormack B, et al. Getting evidence into practice: the role and function of facilitation. J Adv Nurs. 2002;37(6):577-588. <https://doi.org/10.1046/j.1365-2648.2002.02126.x>

17. Helfrich C, Damschroder L, Hagedorn H, Daggett G, Sahay A, Ritchie M, et al. A critical synthesis of literature on the Promoting Action on Research Implementation in Health Services (PARIHS) framework. Implement Sci. 2010;5(1):82. <https://doi.org/10.1186/1748-5908-5-82>

18. Kramer TL, Burns BJ. Implementing Cognitive Behavioral Therapy in the real world: a case study of two mental health centers. Implement Sci. 2008;3(1):14. <https://doi.org/10.1186/1748-5908-3-14>

19. Nagykaldi Z, Mold JW, Aspy CB. Practice facilitators: a review of the literature. Fam Med. 2005;37(8):581-588. <https://doi.org/10.3122/jabfm.19.5.506>

20. Rycroft-Malone J, Kitson A, Harvey G, McCormack B, Seers K, Titchen A, et al. Ingredients for change: revisiting a conceptual framework. Qual Saf Health Care. 2002;11(2):174-180.

21. Rycroft-Malone J, Harvey G, Seers K, Kitson A, McCormack B, Titchen A. An exploration of the factors that influence the implementation of evidence into practice. J Clin Nurs. 2004;13(8):913-924. <https://doi.org/10.1111/j.1365-2702.2004.01007.x>

22. Shirey MR. Evidence-based practice: how nurse leaders can facilitate innovation. Nurs Adm Q. 2006;30(3):252-265.

23. Stetler CB, Legro MW, Rycroft-Malone J, Bowman C, Curran G, Guihan M, et al. Role of "external facilitation" in implementation of research findings: a qualitative evaluation of facilitation experiences in the Veterans Health Administration. Implement Sci. 2006;1:23. <https://doi.org/10.1186/1748-5908-1-23>

24. Sullivan G, Blevins D, Kauth MR. Translating clinical training into practice in complex mental health systems: toward opening the 'black box' of implementation. Implement Sci. 2008;3:33. <https://doi.org/10.1186/1748-5908-3-33>

25. Thompson GN, Estabrooks C, Degner LF. Clarifying the concepts in knowledge transfer: a literature review. J Adv Nurs. 2006;53(6):691-701. <https://doi.org/10.1111/j.1365-2648.2006.03775.x>

26. Curran G, Mukherjee S, Allee E, Owen R. A process for developing an implementation intervention: QUERI Series. Implement Sci. 2008;3(1):17. <https://doi.org/10.1186/1748-5908-3-17>

27. Cheater FM, Hearnshaw H, Baker R, Keane M. Can a facilitated programme promote effective multidisciplinary audit in secondary care teams? an exploratory trial. Int J Nurs Stud. 2005;42(7):779-791. <https://doi.org/10.1016/j.ijnurstu.2004.11.002>

28. Kitson A, Harvey G, McCormack B. Enabling the implementation of evidence based practice: a conceptual framework. Qual Health Care. 1998;7(3):149-158. <https://doi.org/10.1136/qshc.7.3.149>
