# Supplementary material for: Facilitating implementation of primary care mental health over time and across organizational contexts: a qualitative study of role and process
Source: BMC Health Serv Res. 2023 Jun 1;23:565. doi: 10.1186/s12913-023-09598-y (PMC10233920; doi:10.1186/s12913-023-09598-y)
Supplement: Supplementary file 2 — Additional file 2. Debriefing Interviews Coding Template. [file 12913_2023_9598_MOESM2_ESM.docx]

**Additional file 2**

**Debriefing Interviews Coding Template**

**Network # _______ Site: _________________ Quarter #: _____ Coder: ___________________**

| **Facilitation Activity Codes/Who was involved** | **Facilitator/ Date** | **Do/**  **Enable** | **Communication Method(s)** | ***Focus** | **Comments** |
| --- | --- | --- | --- | --- | --- |
| 1. Task Orientation |  |  |  |  |  |
| 2. Problem identification/understand current methods/context |  |  |  |  |  |
| 3. Baseline data |  |  |  |  |  |
| 4. On-going Data & Monitoring |  |  |  |  |  |
| 5. Updates and Feedback |  |  |  |  |  |
| 6. Goal setting/priorities |  |  |  |  |  |
| 7. Action/implementation plans |  |  |  |  |  |
| 8. Strategy/policy development |  |  |  |  |  |
| 9. Clinical education |  |  |  |  |  |
| 10. Change skills education |  |  |  |  |  |
| 11. Marketing education |  |  |  |  |  |
| 12. Marketing |  |  |  |  |  |
| 13. Engaging stakeholders/buy-in |  |  |  |  |  |
| 14. Overcoming resistance to change |  |  |  |  |  |
| 15. Problem-solving |  |  |  |  |  |
| 16. Technical assistance/non-IT |  |  |  |  |  |
| 17. IT |  |  |  |  |  |
| 18. Networking/peers |  |  |  |  |  |
| 19. Networking/experts |  |  |  |  |  |
| 20. Managing team processes |  |  |  |  |  |
| 21. Developing shared vision/consensus  building: |  |  |  |  |  |
| 22. Intercedes/liaises … |  |  |  |  |  |
| 23. Adapt to local context |  |  |  |  |  |
| 24. Organizational change/unspecified |  |  |  |  |  |
| 25. Organization change/structural |  |  |  |  |  |
| 26. Organizational change/culture |  |  |  |  |  |
| 27. Providing support |  |  |  |  |  |
| 28. Administrative Tasks |  |  |  |  |  |
| 29. Hiring internal regional facilitator |  |  |  |  |  |
| 30. Hiring ICP |  |  |  |  |  |
| 31. Pulling back/letting sites take lead |  |  |  |  |  |
| 32. Attended regional meetings |  |  |  |  |  |
| 33. Presented at regional meetings |  |  |  |  |  |
| 34. Organized regional meetings |  |  |  |  |  |
| 35. Attended national meetings |  |  |  |  |  |
| 36. Presented at national meetings |  |  |  |  |  |
| 37. Regional spread/clinical content |  |  |  |  |  |
| 38. Regional spread/facilitation methods |  |  |  |  |  |
| 39. National spread/clinical content |  |  |  |  |  |
| 40. National spread/facilitation methods |  |  |  |  |  |
| 41. Facilitator continuing education |  |  |  |  |  |
| 43. Miscellaneous/Other |  |  |  |  |  |

*Level of focus ratings: Central/High; Moderate; Minor
